# New histopathological and molecular findings in gynecological cancers

**DOI:** 10.1002/ijgo.70283

**Published:** 2025-07-30

**Authors:** W. Glenn McCluggage

**Affiliations:** ^1^ Department of Pathology Belfast Health and Social Care Trust Belfast UK

**Keywords:** cervix, endometrium, female genital tract, gynecological cancer, pathology, tubo‐ovarian, vagina, vulva

## Abstract

This review covers significant developments in the pathological classification of gynecological tumors in recent years. Topics covered include The Cancer Genome Atlas (TCGA) Classification of endometrial carcinomas and how to incorporate this into routine reporting, the fact that most synchronous endometrial and ovarian endometrioid carcinomas represent metastasis from the endometrium to the ovary, and the important subject of lymphovascular space invasion in endometrial carcinomas. The categorization of cervical squamous cell carcinomas (SCCs) and adenocarcinomas and vulval and vaginal SCCs into prognostically meaningful HPV‐associated and HPV‐independent types is also discussed. Some “newly” described tumor types are covered, including endometrial and ovarian mesonephric‐like adenocarcinomas, *STK11* adnexal tumors, and a number of uterine mesenchymal neoplasms associated with specific molecular abnormalities. Important molecular events in ovarian sex cord–stromal tumors and other rare adnexal neoplasms are also discussed.

## 1 INTRODUCTION

The 2020 WHO Classification of Female Genital Tract Tumors (5th edition) was published online and in the traditional “Blue Book” in 2020.^1^ Currently, this Classification is being updated and the sixth edition is expected to be published in 2026. This review covers significant recent developments and major changes in the classification of gynecological cancers, some of which emanate from the 2020 WHO Classification and a few others that have followed. Most of the topics discussed herein cannot be covered in detail and the reader is referred to the key references provided. New technologies, such as single‐cell technologies, liquid biopsies, ascitic fluid profiling, molecular cytopathology, and artificial intelligence, are likely to play an increasing role but these are not discussed in this review.

## 2 THE CANCER GENOME ATLAS (TCGA) MOLECULAR CLASSIFICATION OF ENDOMETRIAL CARCINOMAS

As early as 1983, Bokhman proposed that there were two types of endometrial carcinoma, type I and type II.^2^ Broadly speaking, type I carcinomas (prototypically endometrioid‐type) arise in perimenopausal or early postmenopausal women, are low‐grade, typically early‐stage neoplasms arising on a background of atypical hyperplasia and are positive with hormone receptors. Type II carcinomas (prototypically serous‐type) arise in elderly postmenopausal women, are high‐grade, typically advanced‐stage neoplasms arising in atrophic endometrium, and are negative with hormone receptors. However, although useful as a broad concept, it was always clear that there is significant overlap in the clinical and pathological features in many individual tumors and the Bokhman classification never gained widespread acceptance among pathologists and was never used in pathological reporting.

The current 2020 WHO Classification of endometrial carcinomas,^1^ like prior classifications, is based on morphology. In practice, pathologists often use immunohistochemical markers to assist in classifying problematic neoplasms. However, especially with “high‐grade” endometrial carcinomas (serous, clear cell, grade 3 endometrioid, mixed, undifferentiated/dedifferentiated carcinoma and carcinosarcoma), but not exclusively, there is significant inter‐observer variation even among expert gynecological pathologists.^3^ For example, in one study, three gynecological pathologists examined 56 endometrial carcinomas previously diagnosed as high‐grade and in 20 of 56 (35.8%) there was a major disagreement, including no consensus regarding the tumor type or even whether a component of high‐grade carcinoma was present.^3^

In 2013, the seminal TCGA study of 373 endometrial carcinomas was published; the study only included endometrioid, serous, and mixed endometrioid and serous carcinomas with no other high‐grade carcinomas and employed a variety of modalities, including exome sequencing, somatic copy number alteration, whole genome sequencing, mRNA expression, protein expression, microRNA expression, and DNA methylation.^4^ The study for the first time revealed that endometrial carcinoma consists of four intrinsic molecular types: *POLE* mutated (*POLE*mut)/ultramutated; mismatch repair (MMR) deficient (MMRd)/microsatellite instability‐high (MSI‐H)/hypermutated; p53 abnormal (p53abn)/copy number high; and copy number low/no specific molecular profile (NSMP). It was demonstrated that the four molecular types are of prognostic significance and this has been confirmed in multiple subsequent studies with *POLE*mut tumors having the best prognosis (even though they are often high‐grade and sometimes exhibit substantial lymphovascular space invasion [LVSI], parameters that would typically result in adjuvant treatment) and p53abn the worst, with the other two groups having an intermediate prognosis.^5^, ^6^, ^7^, ^8^, ^9^, ^10^, ^11^, ^12^, ^13^, ^14^, ^15^, ^16^ These molecular groups also form the basis for personalized treatment of endometrial carcinomas based on the molecular classification, for example, de‐escalation of treatment in *POLE*mut tumors, immune checkpoint inhibitors in MMRd and *POLE*mut neoplasms, and chemotherapy in p53abn, while in NSMP tumors, which are hormone receptor positive, hormonal therapy may be an option.^5^, ^6^, ^7^, ^8^, ^9^, ^10^, ^11^, ^12^, ^13^, ^14^, ^15^, ^16^ Importantly, studies have shown that TCGA classification also has prognostic and predictive significance in other endometrial carcinomas, such as carcinosarcoma, clear cell carcinoma, undifferentiated/dedifferentiated carcinoma, and neuroendocrine carcinoma.^17^, ^18^, ^19^

In a recent population‐based study, the percentages within the different TCGA groups were as follows: *POLE*mut 7.3%; MMRd 24%; p53abn 23.7%; and NSMP 45%.^20^ Of the tumors, 6.5% were “multiple‐classifiers” reflecting the fact that *POLE*mut or MMRd endometrial carcinomas may exhibit secondary mutations, for example in *TP53*, due to their ultramutated or hypermutated phenotype. Studies have shown that these neoplasms should be assigned to the molecular group with the best outcome.^21^, ^22^

**FIGURE 1:**
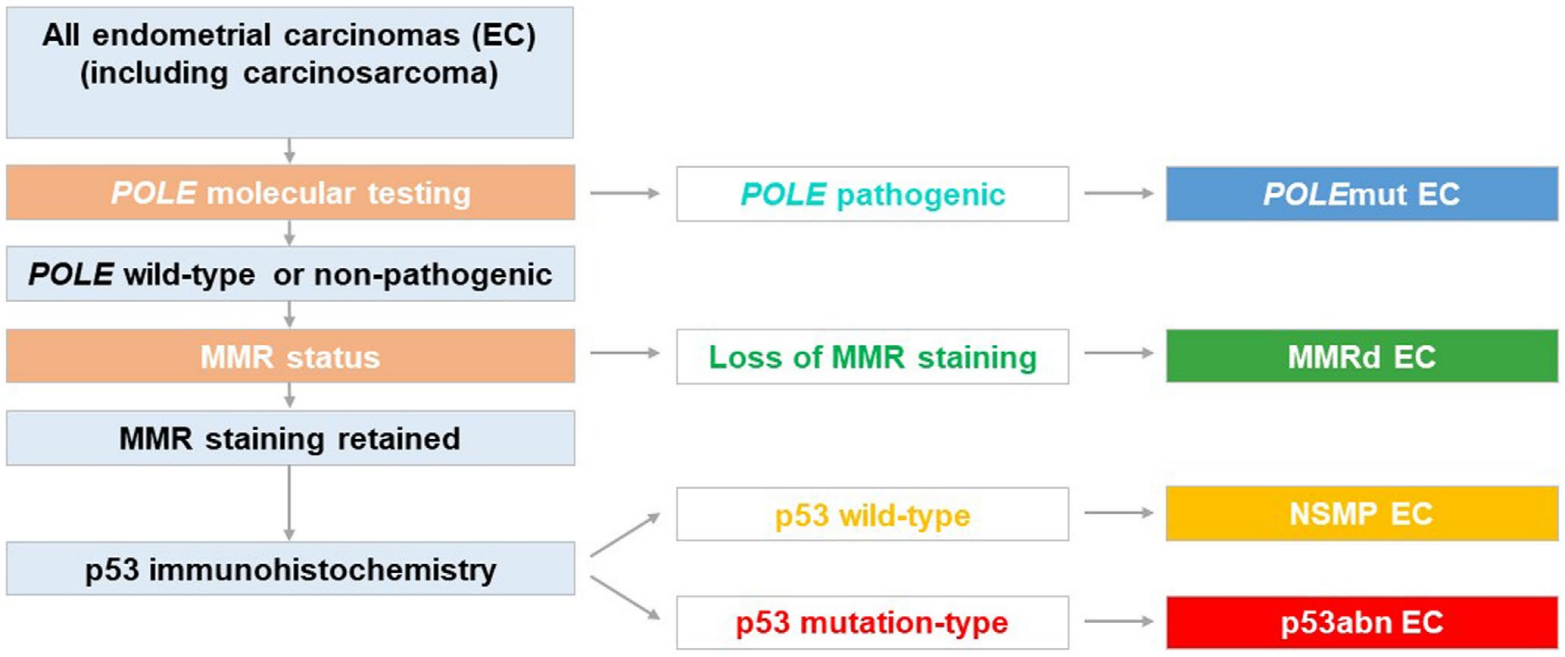
Surrogate ProMisE (Proactive Molecular Risk Classifier for Endometrial Cancer) Classifier for molecular classification of all endometrial carcinomas (regardless of histological type).

Since the original TCGA publication, there has been extensive debate about how best to incorporate full molecular testing or a surrogate of this into the routine reporting of endometrial carcinomas and whether molecular classification is required in all cases. TCGA Classification has been incorporated into various international guidelines including the ESGO/ESTRO/ESP guidelines,^23^ the 2020 WHO Classification,^1^ and the International Federation of Gynecology and Obstetrics (FIGO) 2023 endometrial carcinoma staging system.^24^ While molecular classification can be performed solely by molecular testing using next‐generation sequencing (NGS) panels, which include *POLE*, *TP53*, and MSI analyses, a simplified surrogate classifier, referred to as the ProMisE (Proactive Molecular Risk Classifier for Endometrial Cancer) classifier, is also widely used.^6^, ^8^, ^9^, ^12^ This requires MMR protein and p53 immunohistochemical staining as well as targeted *POLE* molecular testing (Figure 1).

Molecular classification can be performed on biopsy or resection material. The advantage of performing on biopsy specimens is that they are typically better fixed and often comprise a relatively pure tumor population providing higher‐quality material for immunohistochemistry and molecular testing. Immunohistochemistry and NGS analyses are affected by poor fixation and, as a result, are often less than optimal in hysterectomy specimens. Another advantage of performing all the testing on biopsies is that surgical management can potentially be tailored if the molecular group is known preoperatively. A final advantage of testing biopsies is that all the histological, immunohistochemical and molecular studies are available when reporting the hysterectomy specimen, allowing for a comprehensive integrated report. This facilitates prompt decision‐making regarding adjuvant treatment at tumor board meetings.

The absence of a surrogate immunohistochemical marker for predicting *POLE* mutations necessitates molecular testing for TCGA classification. This results in increased costs and potentially turnaround time, and many institutions do not have ready access to *POLE* testing. Molecular classification does not typically change therapeutic approaches in advanced‐stage (III/IV) patients, so *POLE* testing may not be needed; however, even in these cases, the presence of a *POLE* mutation may be indicative of an improved prognosis or response to immunotherapy and a potentially “salvageable” neoplasm and is useful information for the patient. There is a significant group of endometrial carcinomas (grade 1/2 endometrioid, MMR proficient, p53 wild‐type, stage IA, estrogen receptor (ER) positive, no substantial LVSI) where adjuvant treatment will not be given and it could be argued that in these cases *POLE* testing is not necessary. Applying these criteria, Talhouk et al.^9^ found that *POLE* testing could be avoided in 55% of biopsy specimens and 38% of hysterectomy specimens. *POLE* testing should be performed on all MMRd and p53abn endometrial carcinomas in order not to miss a double‐classifier that would fall into the *POLE*mut group.

A challenge is to identify features that are of prognostic and predictive significance in the most common NSMP group. This comprises a heterogeneous group with good prognosis low‐grade endometrioid carcinomas, high‐grade endometrioid carcinomas, and aggressive tumor types, such as clear cell carcinoma, mesonephric‐like adenocarcinoma, and gastric‐type adenocarcinoma. In this group, hormone receptor positivity may be of prognostic and therapeutic value since the ER and progesterone receptor (PR) are usually positive in low‐grade endometrioid carcinomas while the aggressive tumor types just listed are typically negative. Tumor grading is also of most prognostic value in the NSMP group.^25^

It is also worth making the point that molecular classification will always be complementary to traditional pathological parameters since features such as tumor grade, depth of myometrial invasion, LVSI, and cervical and nodal involvement, which are prognostically and therapeutically significant, can only be identified on morphological examination.

## 3 SYNCHRONOUS ENDOMETRIAL AND OVARIAN ENDOMETRIOID CARCINOMAS

It is not rare for a patient to have an endometrioid carcinoma in the endometrium and one, or occasionally both, ovaries. Traditionally, a combination of pathological parameters was used by pathologists to distinguish between synchronous independent primaries and metastasis usually from the endometrium to the ovary. It was long considered that, especially when both the endometrial and ovarian neoplasms are low‐grade, most of these represent dual independent primaries and the prognosis has been assumed to be good, although there are few studies with long‐term follow‐ups. Recent studies have demonstrated that most, but not all, endometrioid neoplasms involving both the endometrium and ovary are clonal, likely representing metastasis from the endometrium to the ovary.^26^, ^27^, ^28^ Thus, there is a dilemma in that, although molecularly these are clonal neoplasms and most represent stage IIIA endometrial carcinomas, the prognosis is thought to be good. There is potential for overtreatment with the unnecessary administration of adjuvant therapy. In WHO 2020 and FIGO 2023, it is recommended that management (generally conservative management without adjuvant therapy) should be as for synchronous neoplasms when the following four criteria are met: (1) both tumors are low‐grade; (2) less than 50% myometrial invasion; (3) no involvement of any other site; and (4) absence of substantial LVSI at any location. In FIGO 2023, such neoplasms are staged as IA3. However, even here there are some inconsistencies between FIGO and WHO, in that bilateral ovarian involvement is not included in the criteria for synchronous neoplasms in FIGO 2023 but is allowable in the 2020 WHO classification.

## 4 LYMPHOVASCULAR SPACE INVASION IN ENDOMETRIAL CARCINOMAS

LVSI has long been regarded as an important prognostic parameter in endometrial carcinoma and has gained increasing interest in recent years due to an expanding body of evidence of its independent prognostic value, especially when the presence of LVSI is quantified. A recent review has covered many aspects of LVSI in endometrial carcinoma.^29^ A key strength of LVSI as a prognostic parameter is that it can be detected on routine microscopic examination, without ancillary tests, and thus can be used in low‐resource settings. A weakness, however, is the lack of uniformly applied criteria for the assessment and quantification of LVSI, resulting in inter‐observer variation in diagnosis.^29^, ^30^ These difficulties are compounded by artifacts and other morphological features that may mimic LVSI (commonly referred to as pseudo‐LVSI). Despite these issues, multiple studies have shown that LVSI, especially substantial or extensive LVSI, is strongly associated with lymph node metastasis and is an independent risk factor for nodal recurrence and distant metastasis.^29^, ^31^, ^32^, ^33^ Consequently, the presence of substantial LVSI has become an important consideration in formulating adjuvant treatment recommendations in patients with endometrial carcinoma. This has been incorporated into management guidelines, such as ESGO/ESTRO/ESP guidelines^23^ and into the recent FIGO 2023 staging system.^24^

Mimics of LVSI include tumor “smearing” and retraction artifacts, which are often secondary to poor fixation. Tumor displacement into vessels may also occur secondary to the use of an intrauterine manipulator during surgery;^34^ these devices are transvaginally inserted into the uterine cavity and allow the surgeon to manipulate the uterus to improve access in the pelvis and identify anatomic structures. However, with manipulator use, tumor cells can be displaced into small and large vascular channels, slit‐like artefactual spaces within the myometrium (which are caused by the manipulator), lumina of the fallopian tubes or beyond. Clues to recognizing this form of pseudo‐LVSI are that the degree of vascular involvement is often greater than would be expected based on the grade and stage of the tumor (for example, in a low‐grade endometrioid carcinoma with minimal myometrial invasion, substantial LVSI would not be expected) and that the tumor emboli often preferentially involve large caliber blood vessels. The microcystic elongated and fragmented (MELF) pattern of myometrial invasion is associated with LVSI but can also mimic true LVSI.^35^

One major problem is that there are no standardized criteria for substantial LVSI resulting in obvious problems. FIGO 2023 uses involvement of five or more lymphovascular spaces to define substantial LVSI, as does the 2020 WHO Classification^1^ and the ESGO/ESTRO/ESP management guidelines.^23^ The National Comprehensive Cancer Network (NCCN) guidelines use four or more spaces in at least one H&E slide^36^ and three or more spaces is used in the 2022 International Collaboration on Cancer Reporting,^37^ the 2019 ISGyP Endometrial Cancer Project recommendations,^38^ and the 2023 CAP cancer reporting protocol.^39^ In most of these recommendations, it is not clarified whether the extent of LVSI is based on the maximum involvement in a single tissue section or on the cumulative extent across all tissue sections. These factors result in difficulties in comparability between practices and regions. For example, if one institution has a very rigorous approach and a high threshold for diagnosis of substantial LVSI, a stage drift compared to other centers will develop. This will result in differing outcomes between centers, stage by stage, not due to real differences in patient outcome, but secondary to systematic differences in stage assignment (the so‐called “stage migration effect”). An additional complicating factor is that a recent large study showed that focal and substantial LVSI in endometrial carcinomas were both associated with increased risk of disease progression but were not prognostically distinct suggesting that focal versus no LVSI have different prognostic outcomes and should not be combined into one category.^40^

## 5 “NEW” CATEGORIES OF ENDOMETRIAL AND OVARIAN CARCINOMA

In WHO 2020, there were some changes to the classification of ovarian and endometrial carcinomas, including the addition of a few new tumor types.^1^ The category of mesonephric‐like adenocarcinoma (MLA) (discussed below) has been added to the classification of both ovarian and endometrial carcinomas. A new category of gastric (gastrointestinal)‐type mucinous carcinoma of the endometrium has been introduced; these are aggressive neoplasms with similar morphology and immunophenotype to gastric‐type adenocarcinomas of the cervix (discussed later). The molecular features of these tumors has not been investigated in detail.^41^

### 5.1 Mesonephric‐like adenocarcinoma

**FIGURE 2:**
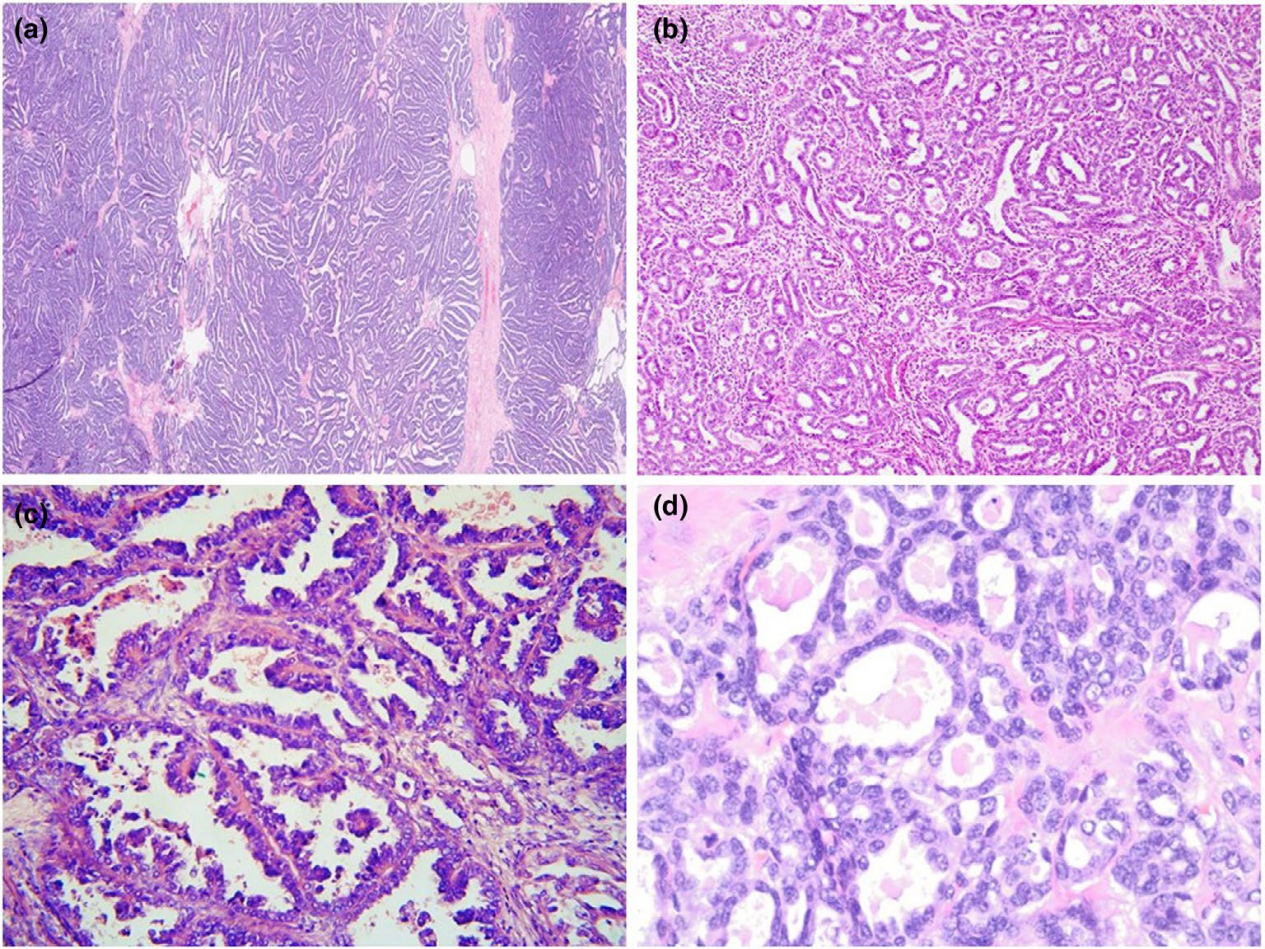
Histological features of MLA. (a) Low‐power showing solid and slit‐like arrangements. (b) Glandular and tubular arrangements. (c) Slit‐like spaces with hobnail cells. (d) Small tubules containing luminal eosinophilic colloid‐like material.

**FIGURE 3:**
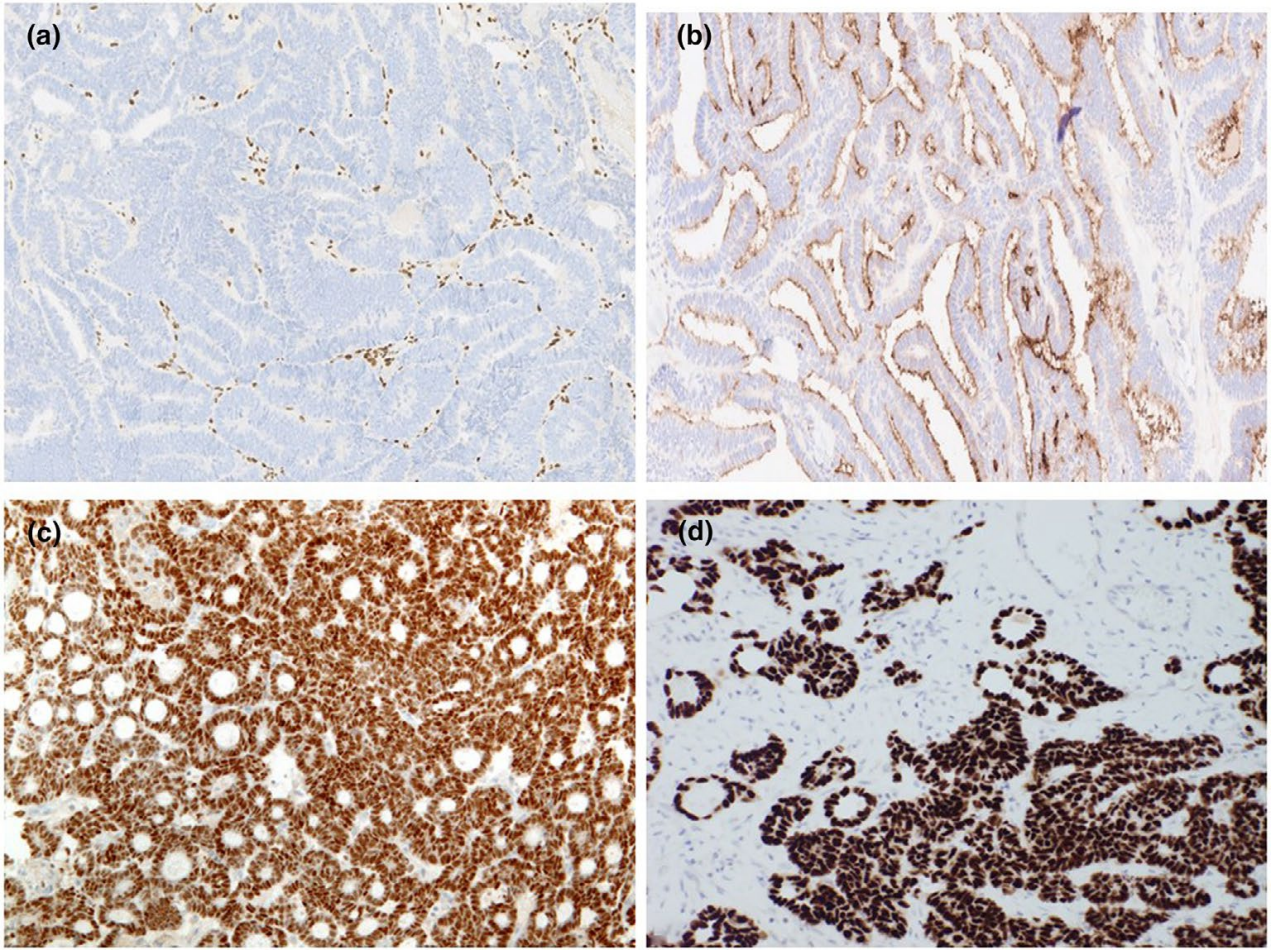
Immunohistochemical features of MLA. Tumor cell nuclei are totally negative with ER (a) and there is luminal positivity with CD10 (b). There is diffuse GATA3 (c) and TTF1 (d) positivity.

The MLA was first reported in 2016 and may arise both in the endometrium and the ovary.^42^, ^43^ These neoplasms are named MLA because they exhibit considerable morphological, immunophenotypic (hormone receptor negative or only focally positive; TTF1 and/ or GATA3 commonly positive), and molecular similarity to true cervical mesonephric adenocarcinomas. They are commonly associated with *KRAS* and, to a lesser extent, *NRAS* mutations.^44^ The term MLA is used because although these neoplasms closely resemble mesonephric adenocarcinomas, other parameters suggest a Müllerian origin and a Müllerian origin has been firmly established. In the uterine corpus, these neoplasms arise from the endometrium and spread into the myometrium while a true mesonephric adenocarcinoma arising from mesonephric remnants would be expected to arise in the myometrium. In the ovary, these neoplasms are commonly associated with endometriosis and like endometrioid and clear cell carcinomas are considered endometriosis‐associated neoplasms. In both the ovary and endometrium, these neoplasms may be associated with other Müllerian neoplasms, such as low‐grade serous and endometrioid carcinomas.^42^, ^43^ Moreover, in both the uterine corpus and ovary, these neoplasms are not associated with mesonephric remnants. Mesonephric‐like carcinosarcomas have also been described, as has an origin in extraovarian endometriosis.^42^, ^43^, ^45^ Figures 2 and 3 show histological images and the immunophenotype of MLA.

Several studies have shown that MLAs of both the endometrium and the ovary are aggressive neoplasms with a propensity for presentation at advanced stage and to develop distant spread, especially to the lungs and other unusual sites.^46^, ^47^, ^48^ MLA are not graded but are regarded automatically as high‐grade. In TCGA molecular classification of endometrial carcinomas, almost all MLAs belong to the NSMP category.

## 6 “NEW” DESCRIBED UTERINE MESENCHYMAL NEOPLASMS

Until relatively recently, almost all uterine sarcomas were considered to represent leiomyosarcomas, low‐grade endometrial stromal sarcomas, undifferentiated sarcomas, or rare “heterologous” sarcomas, such as rhabdomyosarcoma. However, the last decade has witnessed the description of several “new” uterine sarcoma types, such as high‐grade endometrial stromal sarcomas associated with *YWHAE‐NUTM2A/B* or *BCOR* abnormalities, undifferentiated sarcomas associated with *SMARCA4* mutation, and sarcomas associated with neurotrophic tropomyosin receptor kinase (*NTRK*) rearrangements (these predominantly have a cervical location) and *KAT6A/B:: KANSL1* fusions.^49^, ^50^, ^51^, ^52^, ^53^, ^54^, ^55^ Predominantly these neoplasms were discovered using molecular techniques, such as NGS, which have revealed novel diagnostic molecular events. Various other molecular abnormalities have also been reported in uterine sarcomas and with the increasing availability of these molecular techniques it is inevitable that additional “new” entities will be reported in the near future. This will result in diminution of the category of undifferentiated uterine sarcoma. Although many of these entities can be suspected on morphological and immunohistochemical examination, a definitive diagnosis of most of these neoplasms requires molecular confirmation. Since these are rare neoplasms and the testing infrastructure is not available in most pathology laboratories (even those with NGS panels), diagnosis will be facilitated by referral to centers that can undertake the necessary testing and to specialist pathologists.

The last few years has also witnessed the publication of several studies that help to predict the behavior of uncommon uterine mesenchymal lesions, such as perivascular epithelioid cell tumor (PEComa), inflammatory myofibroblastic tumor (IMT), and uterine tumor resembling ovarian sex cord tumors (UTROSCTs).^56^, ^57^, ^58^, ^59^, ^60^

## 7 HPV‐ASSOCIATED AND HPV‐INDEPENDENT CERVICAL CARCINOMAS

The 2014 WHO Classification of cervical SCCs divided these neoplasms into meaningless morphological types, such as keratinizing, non‐keratinizing, basaloid, warty, papillary, squamotransitional, verrucous, and lymphoepithelioma‐like. These represent morphological variations rather than tumor types and, in practice, most pathologists did not use these categories, which suffered from lack of reproducibility and were of no prognostic significance. The 2020 WHO Classification categorizes cervical SCCs into HPV‐associated and HPV‐independent types.^1^ This is a natural and welcome extension of the trend to categorize SCC at many sites into HPV‐associated and HPV‐independent types, for example in the vulva and vagina (see below), and in extragenital sites, such as the head and neck region. At most sites, the division into HPV‐associated and HPV‐independent SCC has prognostic significance with a generally better prognosis for HPV‐associated neoplasms. The prognostic significance in the cervix is not yet well‐established given that HPV‐independent SCCs are uncommon, although there is some evidence that they are more aggressive and present at higher tumor stages;^61^, ^62^, ^63^, ^64^ they were estimated in one study to comprise approximately 2% of cervical SCCs but 7% and 17% in patients over aged 60 and 70 years, respectively.^62^ HPV‐independent cervical SCCs are more likely to arise in older patients, sometimes associated with uterine prolapse, and some are associated with *TP53* mutations.^63^ Given the marked preponderance of HPV‐associated neoplasms, it is controversial as to whether confirmatory studies to confirm an HPV association (most commonly p16 immunohistochemistry but also HPV testing) should be undertaken in all cases, especially since there are currently no management or well‐established prognostic implications. However, since morphology is not always reliable in distinguishing between HPV‐associated and HPV‐independent SCCs (there are some morphological differences in that HPV‐associated SCCs tend to have a basaloid morphology with limited keratinization while HPV‐independent SCCs tend to be keratinizing), an argument can be made for undertaking p16 staining in all cervical SCC and if staining is not block‐type (this pattern of p16 staining being a useful surrogate marker of a high‐risk HPV‐associated SCC), an HPV‐independent neoplasm should be considered and HPV testing undertaken using sensitive molecular techniques. An alternative is to undertake ancillary testing only in those cases where the morphology (keratinizing) raises the possibility of an HPV‐independent neoplasm or when corroborative evidence of an HPV‐associated neoplasm, such as adjacent high‐grade squamous intraepithelial lesion, is absent. The 2020 WHO Classification also includes a category of SCC, not otherwise specified (NOS), to be used in settings where p16 staining or HPV testing is not available.^1^ Given that HPV vaccination programs are now instigated in many high‐income countries, it is clear that HPV‐independent neoplasms will comprise an increasing proportion of cervical SCCs and other neoplasms that may be HPV‐associated or HPV‐independent.

In WHO 2014, as with cervical SCCs, cervical adenocarcinomas were classified based on morphology and were divided into clinically meaningless and poorly reproducible categories. Analogous to cervical SCCs, cervical adenocarcinomas are now categorized in WHO 2020 into HPV‐associated and HPV‐independent types. Most cervical adenocarcinomas are HPV‐associated but a higher percentage than SCC (about 15%–20%) are HPV‐independent.^65^, ^66^, ^67^, ^68^, ^69^, ^70^, ^71^ HPV‐independent cervical adenocarcinomas typically present at higher stage and have a worse prognosis.^65^, ^66^, ^67^, ^68^, ^69^, ^70^, ^71^ The 2020 WHO Classification also divides adenocarcinoma precursor lesions (adenocarcinoma in situ) into HPV‐associated and HPV‐independent types, the latter including gastric‐type adenocarcinoma in situ and atypical lobular endocervical glandular hyperplasia.^72^, ^73^

HPV‐associated adenocarcinomas are typified by easily identifiable mitotic figures and apoptotic bodies and almost always exhibit diffuse block‐type immunoreactivity with p16. Subtypes of HPV‐associated adenocarcinoma include usual type and mucinous type; the former encompasses villoglandular and micropapillary variants and mucinous type encompasses stratified mucin producing carcinoma, intestinal, signet ring, and NOS variants.^1^ HPV‐independent types of cervical adenocarcinoma are the gastric (the most common), mesonephric, and clear cell types.^65^, ^66^, ^67^, ^68^, ^69^, ^70^, ^71^, ^74^ As discussed, these typically have a worse prognosis than HPV‐associated adenocarcinomas and are almost always p16 negative or focally positive (non block‐type immunoreactivity). Of note, serous carcinoma of the cervix is not included in the 2020 WHO Classification. Most tumors previously diagnosed as such represent HPV‐associated cervical adenocarcinomas with papillary/micropapillary architecture and high‐grade nuclear features mimicking serous carcinoma or involvement by a tubo‐ovarian or endometrial serous carcinoma; as such, a diagnosis of a primary cervical serous carcinoma should not be made.

## 8 HPV‐ASSOCIATED AND HPV‐INDEPENDENT VULVAL AND VAGINAL CARCINOMAS

As in the cervix, vaginal and vulval SCCs are now divided into HPV‐associated and HPV‐independent types in WHO 2020.^1^ This terminology replaces those types included in the prior classification, namely keratinizing, non‐keratinizing, papillary, basaloid, warty, and verrucous. The reasons underlying these changes are exactly analogous to those discussed in the section on cervical SCCs. Although primary vaginal SCCs are considerably more uncommon than in the cervix and the vulva, there is convincing evidence that, as at other sites, HPV‐independent neoplasms have a worse prognosis.^75^ It is recommended that the type of vaginal SCC (HPV‐associated or HPV‐independent) be documented on the pathology report. However, as at other sites, a morphological diagnosis of SCC NOS is acceptable when resources required to differentiate between the two, such as p16 immunohistochemistry and HPV testing, are not available.

Similarly in the vulva, traditional histological typing of vulval SCC has been superseded by HPV status as the major determinant of classification. HPV‐independent SCCs have a worse prognosis with significantly worse recurrence‐free and overall survival compared with HPV‐associated SCCs.^76^, ^77^, ^78^, ^79^, ^80^, ^81^ There is also growing evidence that HPV‐independent SCCs are less responsive to radiotherapy and are more likely to recur with close margins. The majority of HPV‐associated SCC exhibit basaloid or warty morphology, while HPV‐independent SCC tend to be keratinizing; however, a significant percentage of cases (15%–20%) show overlapping morphologic features. Although the nature of any adjacent precursor lesion may be useful in helping to determine the HPV status, in practice, ancillary testing (p16 or HPV testing) is necessary given the overlap in morphology. As in the cervix and vagina when HPV status cannot be confidently determined or resources are not available to undertake ancillary testing, a diagnosis of SCC NOS is acceptable, although this is not recommended. Most, but not all, HPV‐independent vulval SCCs are associated with *TP53* mutations.^76^, ^77^, ^78^, ^79^, ^80^, ^81^ However, a proportion is *TP53* wild type and there is growing evidence that these latter neoplasms have an intermediate prognosis between HPV‐associated SCCs (best prognosis) and HPV‐independent *TP53* mutated neoplasms (worst prognosis);^82^ as such, p53 immunohistochemistry or *TP53* molecular testing may be useful in HPV‐independent SCCs. Precursor lesions of vulval SCCs are now also classified as HPV‐associated or HPV‐independent.^1^, ^83^

Primary vaginal adenocarcinomas are extremely rare and there have also been changes to the classification of these with the description of new entities, such as HPV‐associated and gastric‐type adenocarcinomas,^84^, ^85^ both of which may arise in adenosis. The gastric‐type are aggressive primary vaginal adenocarcinomas, morphologically and immunophenotypically identical to their cervical counterparts.

## 9 NEW DEVELOPMENTS IN OVARIAN SEX CORD–STROMAL TUMORS AND MISCELLANEOUS NEOPLASMS

Ovarian sex cord–stromal tumors is one area of pathology where significant advances have been made in recent years, especially in elucidation of the underlying molecular events. Sex cord–stromal tumors represent an uncommon and heterogeneous group of neoplasms that, when they exhibit classical morphology, are relatively easy to diagnose. However, there may be considerable morphological overlap between the different tumor types, and immunohistochemistry, while useful in confirming a sex cord–stromal tumor, is of minimal value in distinguishing between the different tumor types.

Recent significant advances regarding molecular events (Table 1) include the demonstration that adult granulosa cell tumors contain somatic *FOXL2* mutations in well over 90% of cases,^86^, ^87^, ^88^, ^89^ while a very large percentage of moderately and poorly differentiated Sertoli–Leydig cell tumors (SLCTs) contain *DICER1* mutations; these may be somatic or germline, the latter signifying DICER1 syndrome.^88^, ^89^, ^90^ It has also been demonstrated that well‐differentiated SLCTs represent a fundamentally different tumor type to moderately and poorly differentiated and is not associated with *DICER1* variants.^91^ Other studies have elucidated the molecular events in several other tumor types within the sex cord–stromal category. For example, a microcystic stromal tumor contains *CTNNB1* or less frequently *APC* mutations and is occasionally an extracolonic manifestation of familial adenomatous polyposis,^92^, ^93^ while a sclerosing stromal tumor is associated with *FHL2:: GLI2* fusions.^94^ In problematic cases, demonstration of the appropriate molecular abnormality assists in tumor classification.

**TABLE 1:** Molecular events in ovarian sex cord–stromal tumors and other uncommon adnexal or para‐adnexal neoplasms.

Tumor	Recurrent molecular events (useful in diagnosis)	Other molecular events (may be useful in prognostication)	Tumor syndrome
AGCT	*FOXL2* (somatic missense C134W mutation)	*TP53*, *TERT*, *CDKN2A*, *KMT2D*, *MED12*	None
JGCT	None	*TERT*, *AKTC*, *GNAS*, *KMT2C*	Ollier disease and Maffucci syndrome
Steroid cell tumor	None	Genomic instability in malignant tumors	None
SCTAT	*STK11* mutations in Peutz ‐Jeghers associated cases		Peutz‐Jeghers syndrome
SLCT	*DICER1* variants (except well‐differentiated SLCT) *FOXL2* variants in some tumors in older patients		DICER1 syndrome
Sclerosing stromal tumor	*FHL2*: *GLI2* fusion		None
MST	*CTNNB1* and *APC* mutations		Familial adenomatous polyposis
SCCOHT	*SMARCA4* mutations		Familial SCCOHT
*STK11* adnexal tumor	*STK11* mutations and other *STK11* aberrations		Peutz‐Jeghers syndrome

Abbreviations: AGCT, adult granulosa cell tumor; JGCT, juvenile granulosa cell tumor; MST (microcystic stromal tumor); SCCOHT, small cell carcinoma of the ovary of hypercalcemic type; SCTAT, sex cord tumor with annular tubules; SLCT, Sertoli Leydig cell tumor.

Small cell carcinoma of the ovary of hypercalcemic type (SCCOHT), which is included in the category of miscellaneous ovarian neoplasms in WHO 2020, has been shown to be characterized by deleterious germline or somatic mutations in a single gene *SMARCA4* ^95^, ^96^, ^97^ in well over 90% of cases. *SMARCA4* is part of the SWI/SNF complex, which is implicated in the pathogenesis of a growing number of malignancies.^98^ Demonstration of this mutation and/or loss of immunohistochemical staining with SMARCA4 (BRG1) antibody may, in the correct morphological context, be crucial in the diagnosis of this highly aggressive neoplasm.^99^, ^100^, ^101^ It is recommended that all patients diagnosed with SCCOHT should be referred for germline *SMARCA4* mutation testing.^102^

When diagnosing some of these uncommon neoplasms, the pathologist should raise the possibility of a germline mutation, for example *DICER1* in SLCT or *SMARCA4* in SCCOHT and recommend genetic referral and germline testing. It is also worth noting that given the rarity of some of these neoplasms and the overlap in morphology between them, many will benefit from referral for a specialist opinion which, as well as hopefully ensuring a correct diagnosis, facilitates the accrual of case series.^103^

## 10 *STK11* ADNEXAL TUMOR

The *STK11* adnexal tumor is a rare, recently described neoplasm that may be associated with Peutz–Jeghers syndrome (PJS). In the original report of this neoplasm, 47% of patients were known to have PJS with *STK11* germline mutations.^104^ Tumors have occurred in patients with a wide age range (16–74 years) and predominately originate in the para‐adnexal soft tissues, sometimes with secondary involvement of the fallopian tube and ovary.^104^, ^105^ These are aggressive tumors with frequent metastases and recurrences.

*STK11* adnexal tumors are morphologically heterogeneous with a wide range of different architectural patterns and cytological features. While their immunohistochemical profile is non‐specific, they typically have a “polyphenotypic” immunophenotype, often with positivity for cytokeratins, hormone receptors, mesothelial, and sex‐cord markers. Due to their location and heterogeneous morphology and immunoprofile, *STK11* adnexal tumors have a broad differential diagnosis, including various Müllerian carcinomas (especially endometrioid carcinoma), mesothelioma, female adnexal tumor of Wolffian origin (FATWO), and sex cord–stromal tumors.

Currently, the diagnosis of an *STK11* adnexal tumor requires molecular confirmation of *STK11* inactivation/alterations. The reporting pathologist must have a high index of suspicion to think of the diagnosis and instigate molecular testing. It is likely that many tumors previously reported as malignant FATWOs represent *STK11* adnexal tumors. When making a diagnosis of *STK11* adnexal tumor, the pathologist should mention the possibility of PJS on the pathology report if the patient is not already known to have this syndrome.

## AUTHOR CONTRIBUTIONS

WGM is responsible for the design and writing of the paper, and agrees to be accountable for all aspects of the published paper.

## CONFLICT OF INTEREST STATEMENT

The author has no conflicts of interest.

## Data Availability

Data sharing is not applicable to this article as no new data were created or analyzed in this study.
